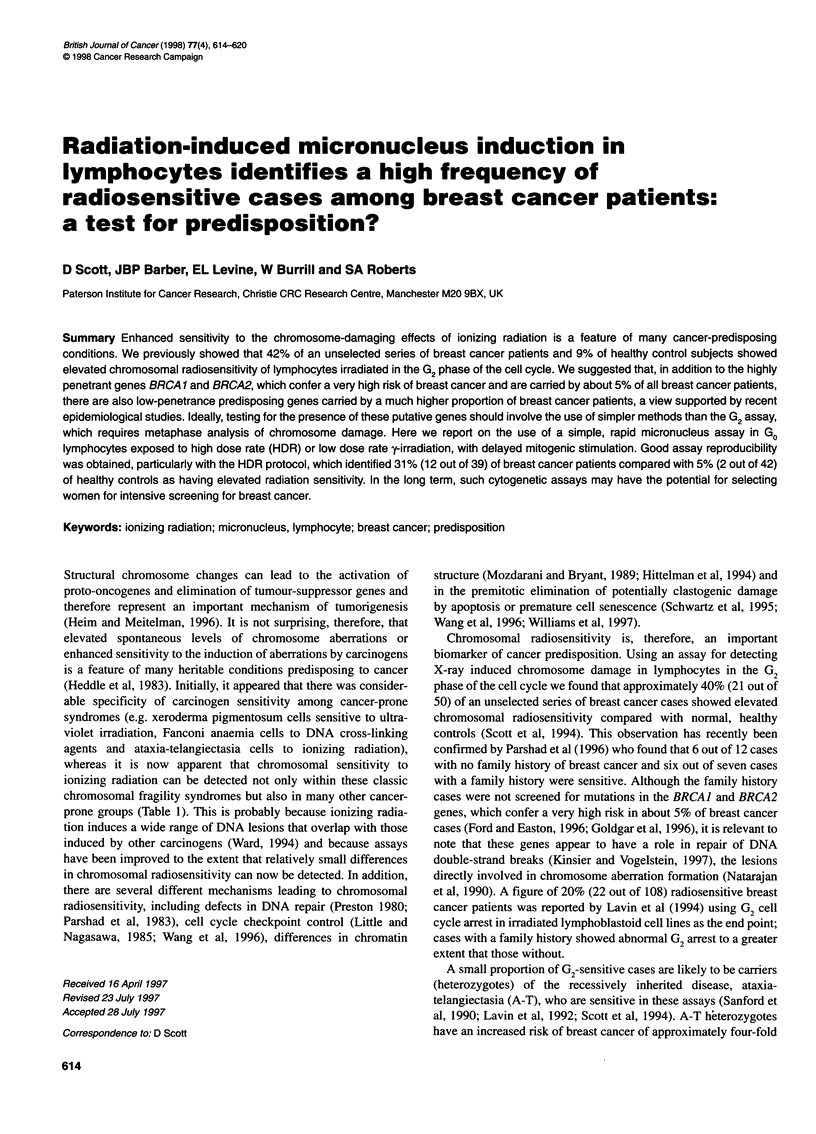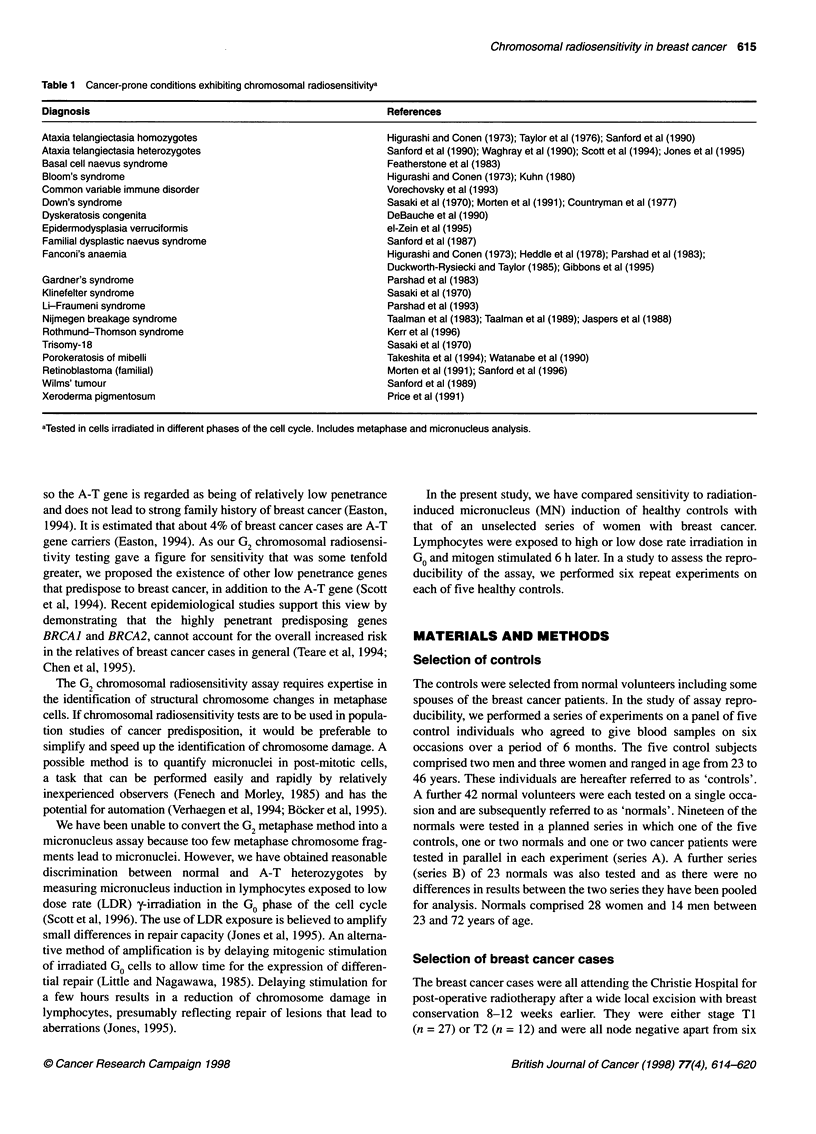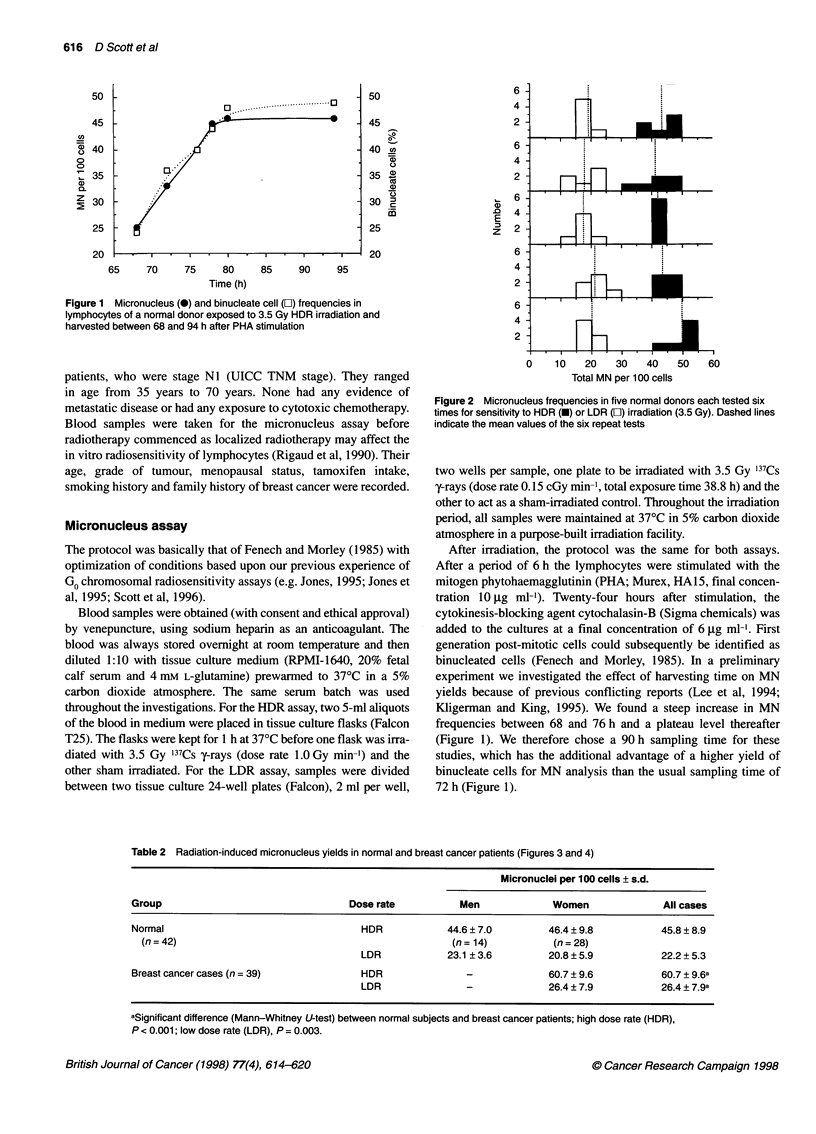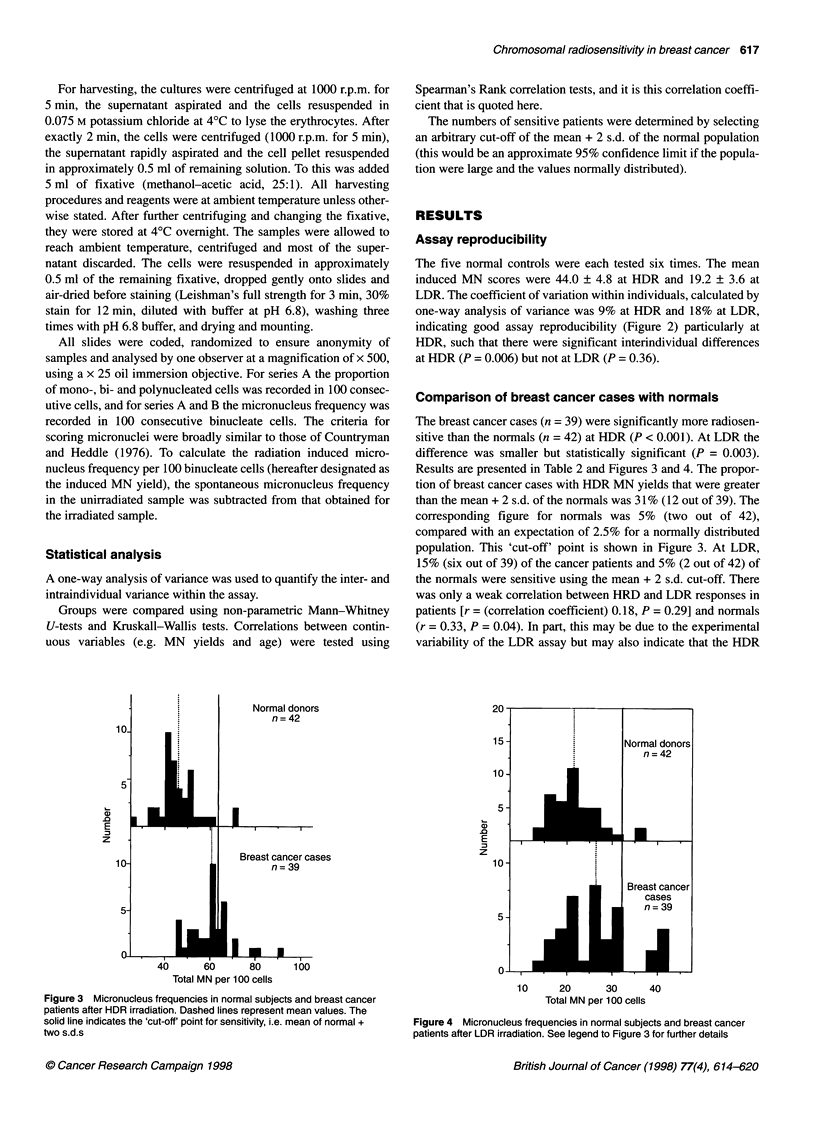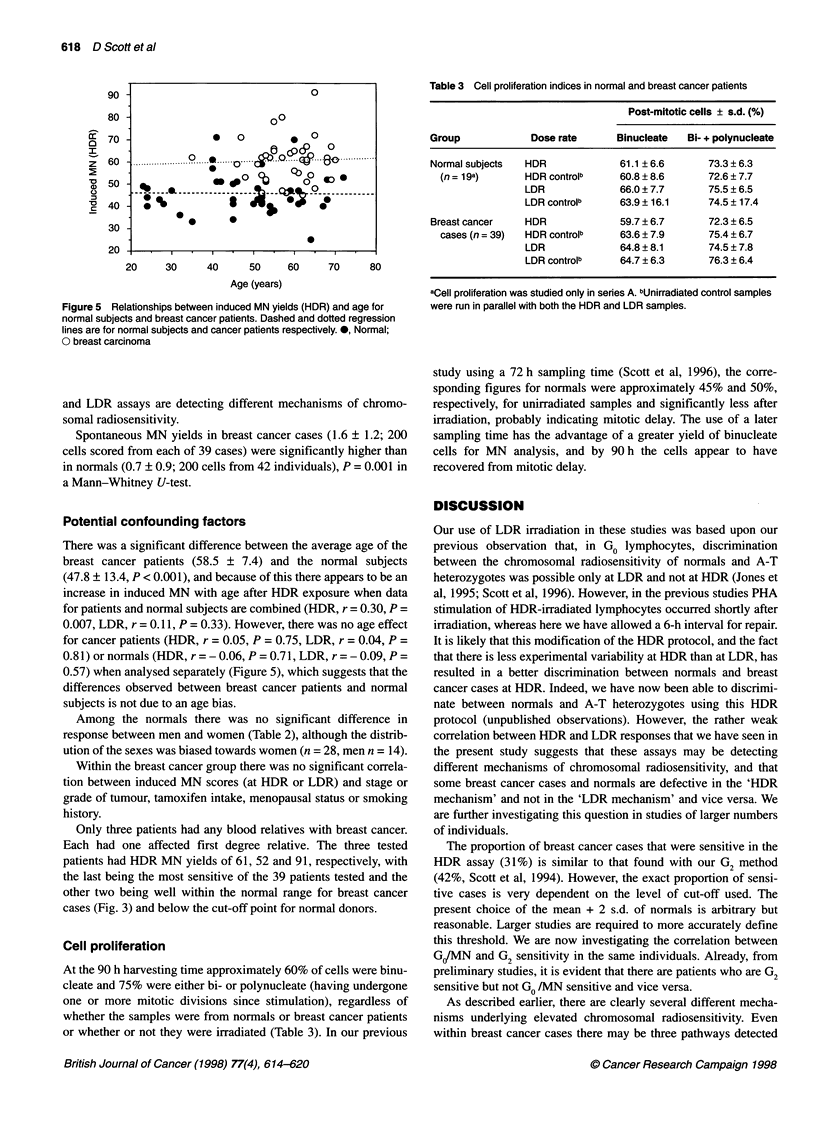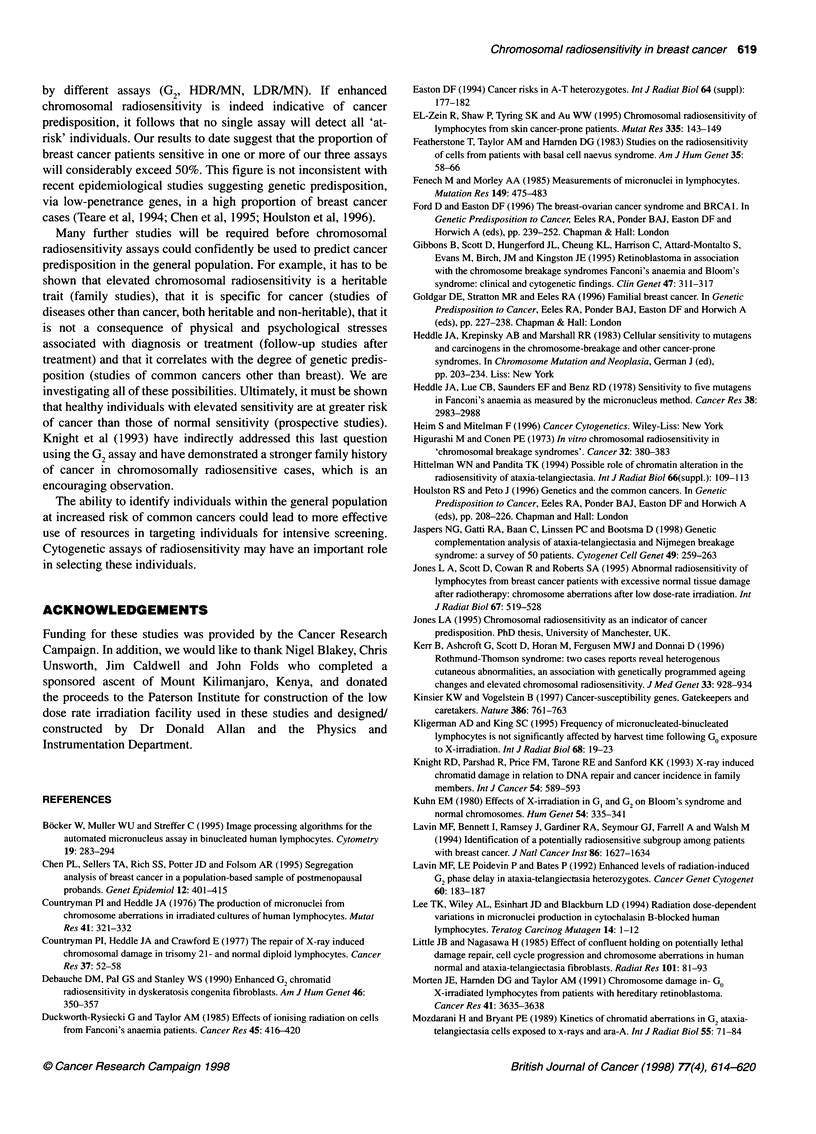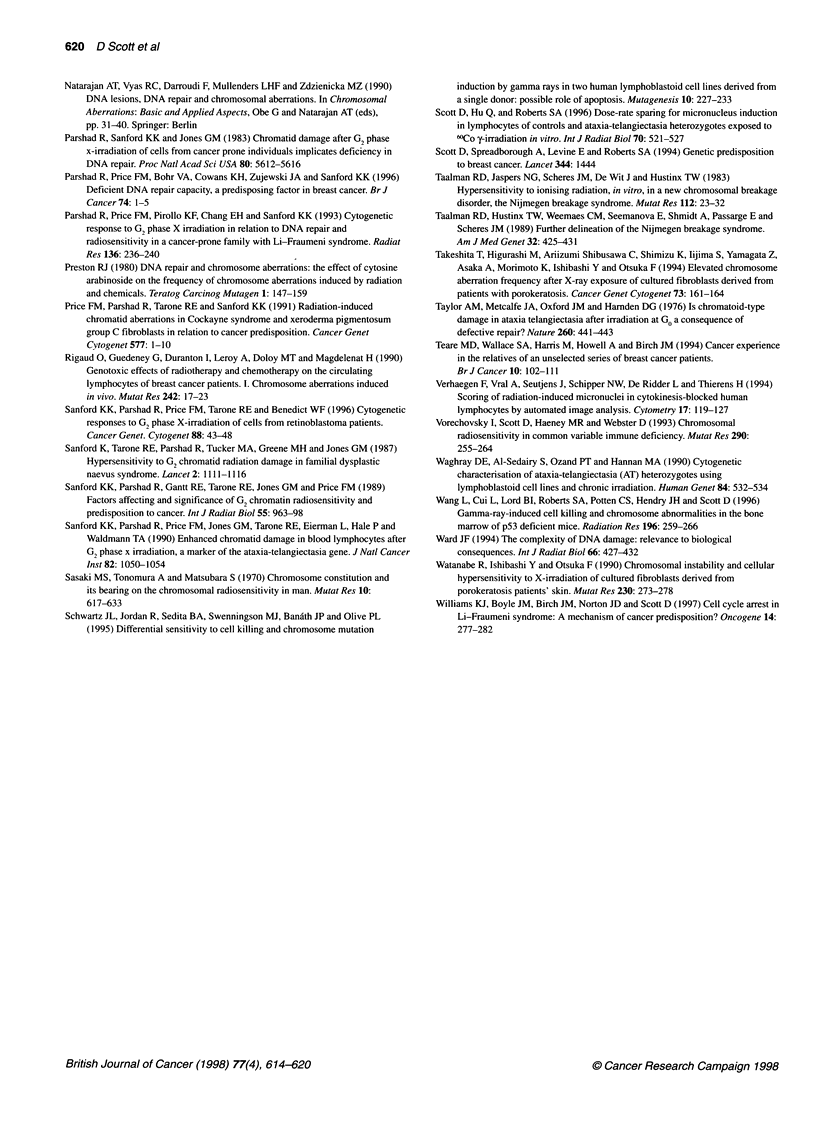# Radiation-induced micronucleus induction in lymphocytes identifies a high frequency of radiosensitive cases among breast cancer patients: a test for predisposition?

**DOI:** 10.1038/bjc.1998.98

**Published:** 1998-02

**Authors:** D. Scott, J. B. Barber, E. L. Levine, W. Burrill, S. A. Roberts

**Affiliations:** Paterson Institute for Cancer Research, Christie CRC Research Centre, Manchester, UK.

## Abstract

Enhanced sensitivity to the chromosome-damaging effects of ionizing radiation is a feature of many cancer-predisposing conditions. We previously showed that 42% of an unselected series of breast cancer patients and 9% of healthy control subjects showed elevated chromosomal radiosensitivity of lymphocytes irradiated in the G2 phase of the cell cycle. We suggested that, in addition to the highly penetrant genes BRCA1 and BRCA2, which confer a very high risk of breast cancer and are carried by about 5% of all breast cancer patients, there are also low-penetrance predisposing genes carried by a much higher proportion of breast cancer patients, a view supported by recent epidemiological studies. Ideally, testing for the presence of these putative genes should involve the use of simpler methods than the G2 assay, which requires metaphase analysis of chromosome damage. Here we report on the use of a simple, rapid micronucleus assay in G0 lymphocytes exposed to high dose rate (HDR) or low dose rate gamma-irradiation, with delayed mitogenic stimulation. Good assay reproducibility was obtained, particularly with the HDR protocol, which identified 31% (12 out of 39) of breast cancer patients compared with 5% (2 out of 42) of healthy controls as having elevated radiation sensitivity. In the long term, such cytogenetic assays may have the potential for selecting women for intensive screening for breast cancer.


British Journal of Cancer (1998) 77(4), 614–620
© 1998 Cancer Research Campaign

### Radiation-induced micronucleus induction in lymphocytes identifies a high frequency of radiosensitive cases among breast cancer patients: a test for predisposition?

**D Scott, JBP Barber, EL Levine, W Burrill and SA Roberts**

Paterson Institute for Cancer Research, Christie CRC Research Centre, Manchester M20 9BX, UK

**Summary** Enhanced sensitivity to the chromosome-damaging effects of ionizing radiation is a feature of many cancer-predisposing conditions. We previously showed that 42% of an unselected series of breast cancer patients and 9% of healthy control subjects showed elevated chromosomal radiosensitivity of lymphocytes irradiated in the $G_2$ phase of the cell cycle. We suggested that, in addition to the highly penetrant genes *BRCA1* and *BRCA2*, which confer a very high risk of breast cancer and are carried by about 5% of all breast cancer patients, there are also low-penetrance predisposing genes carried by a much higher proportion of breast cancer patients, a view supported by recent epidemiological studies. Ideally, testing for the presence of these putative genes should involve the use of simpler methods than the $G_2$ assay, which requires metaphase analysis of chromosome damage. Here we report on the use of a simple, rapid micronucleus assay in $G_0$ lymphocytes exposed to high dose rate (HDR) or low dose rate γ-irradiation, with delayed mitogenic stimulation. Good assay reproducibility was obtained, particularly with the HDR protocol, which identified 31% (12 out of 39) of breast cancer patients compared with 5% (2 out of 42) of healthy controls as having elevated radiation sensitivity. In the long term, such cytogenetic assays may have the potential for selecting women for intensive screening for breast cancer.

**Keywords:** ionizing radiation; micronucleus, lymphocyte; breast cancer; predisposition

Structural chromosome changes can lead to the activation of proto-oncogenes and elimination of tumour-suppressor genes and therefore represent an important mechanism of tumorigenesis (Heim and Meitelman, 1996). It is not surprising, therefore, that elevated spontaneous levels of chromosome aberrations or enhanced sensitivity to the induction of aberrations by carcinogens is a feature of many heritable conditions predisposing to cancer (Heddle et al, 1983). Initially, it appeared that there was considerable specificity of carcinogen sensitivity among cancer-prone syndromes (e.g. xeroderma pigmentosum cells sensitive to ultraviolet irradiation, Fanconi anaemia cells to DNA cross-linking agents and ataxia-telangiectasia cells to ionizing radiation), whereas it is now apparent that chromosomal sensitivity to ionizing radiation can be detected not only within these classic chromosomal fragility syndromes but also in many other cancer-prone groups (Table 1). This is probably because ionizing radiation induces a wide range of DNA lesions that overlap with those induced by other carcinogens (Ward, 1994) and because assays have been improved to the extent that relatively small differences in chromosomal radiosensitivity can now be detected. In addition, there are several different mechanisms leading to chromosomal radiosensitivity, including defects in DNA repair (Preston 1980; Parshad et al, 1983), cell cycle checkpoint control (Little and Nagasawa, 1985; Wang et al, 1996), differences in chromatin structure (Mozdarani and Bryant, 1989; Hittelman et al, 1994) and in the premitotic elimination of potentially clastogenic damage by apoptosis or premature cell senescence (Schwartz et al, 1995; Wang et al, 1996; Williams et al, 1997).

Chromosomal radiosensitivity is, therefore, an important biomarker of cancer predisposition. Using an assay for detecting X-ray induced chromosome damage in lymphocytes in the $G_2$ phase of the cell cycle we found that approximately 40% (21 out of 50) of an unselected series of breast cancer cases showed elevated chromosomal radiosensitivity compared with normal, healthy controls (Scott et al, 1994). This observation has recently been confirmed by Parshad et al (1996) who found that 6 out of 12 cases with no family history of breast cancer and six out of seven cases with a family history were sensitive. Although the family history cases were not screened for mutations in the *BRCA1* and *BRCA2* genes, which confer a very high risk in about 5% of breast cancer cases (Ford and Easton, 1996; Goldgar et al, 1996), it is relevant to note that these genes appear to have a role in repair of DNA double-strand breaks (Kinsier and Vogelstein, 1997), the lesions directly involved in chromosome aberration formation (Natarajan et al, 1990). A figure of 20% (22 out of 108) radiosensitive breast cancer patients was reported by Lavin et al (1994) using $G_2$ cell cycle arrest in irradiated lymphoblastoid cell lines as the end point; cases with a family history showed abnormal $G_2$ arrest to a greater extent that those without.

A small proportion of $G_2$-sensitive cases are likely to be carriers (heterozygotes) of the recessively inherited disease, ataxia-telangiectasia (A-T), who are sensitive in these assays (Sanford et al, 1990; Lavin et al, 1992; Scott et al, 1994). A-T heterozygotes have an increased risk of breast cancer of approximately four-fold

*Received 16 April 1997*
*Revised 23 July 1997*
*Accepted 28 July 1997*

*Correspondence to:* D Scott

**Table 1** Cancer-prone conditions exhibiting chromosomal radiosensitivity[a]

| Diagnosis | References |
|---|---|
| Ataxia telangiectasia homozygotes | Higurashi and Conen (1973); Taylor et al (1976); Sanford et al (1990) |
| Ataxia telangiectasia heterozygotes | Sanford et al (1990); Waghray et al (1990); Scott et al (1994); Jones et al (1995) |
| Basal cell naevus syndrome | Featherstone et al (1983) |
| Bloom's syndrome | Higurashi and Conen (1973); Kuhn (1980) |
| Common variable immune disorder | Vorechovsky et al (1993) |
| Down's syndrome | Sasaki et al (1970); Morten et al (1991); Countryman et al (1977) |
| Dyskeratosis congenita | DeBauche et al (1990) |
| Epidermodysplasia verruciformis | el-Zein et al (1995) |
| Familial dysplastic naevus syndrome | Sanford et al (1987) |
| Fanconi's anaemia | Higurashi and Conen (1973); Heddle et al (1978); Parshad et al (1983); Duckworth-Rysiecki and Taylor (1985); Gibbons et al (1995) |
| Gardner's syndrome | Parshad et al (1983) |
| Klinefelter syndrome | Sasaki et al (1970) |
| Li–Fraumeni syndrome | Parshad et al (1993) |
| Nijmegen breakage syndrome | Taalman et al (1983); Taalman et al (1989); Jaspers et al (1988) |
| Rothmund–Thomson syndrome | Kerr et al (1996) |
| Trisomy-18 | Sasaki et al (1970) |
| Porokeratosis of mibelli | Takeshita et al (1994); Watanabe et al (1990) |
| Retinoblastoma (familial) | Morten et al (1991); Sanford et al (1996) |
| Wilms' tumour | Sanford et al (1989) |
| Xeroderma pigmentosum | Price et al (1991) |

[a]Tested in cells irradiated in different phases of the cell cycle. Includes metaphase and micronucleus analysis.

so the A-T gene is regarded as being of relatively low penetrance and does not lead to strong family history of breast cancer (Easton, 1994). It is estimated that about 4% of breast cancer cases are A-T gene carriers (Easton, 1994). As our $G_2$ chromosomal radiosensitivity testing gave a figure for sensitivity that was some tenfold greater, we proposed the existence of other low penetrance genes that predispose to breast cancer, in addition to the A-T gene (Scott et al, 1994). Recent epidemiological studies support this view by demonstrating that the highly penetrant predisposing genes *BRCA1* and *BRCA2*, cannot account for the overall increased risk in the relatives of breast cancer cases in general (Teare et al, 1994; Chen et al, 1995).

The $G_2$ chromosomal radiosensitivity assay requires expertise in the identification of structural chromosome changes in metaphase cells. If chromosomal radiosensitivity tests are to be used in population studies of cancer predisposition, it would be preferable to simplify and speed up the identification of chromosome damage. A possible method is to quantify micronuclei in post-mitotic cells, a task that can be performed easily and rapidly by relatively inexperienced observers (Fenech and Morley, 1985) and has the potential for automation (Verhaegen et al, 1994; Böcker et al, 1995).

We have been unable to convert the $G_2$ metaphase method into a micronucleus assay because too few metaphase chromosome fragments lead to micronuclei. However, we have obtained reasonable discrimination between normal and A-T heterozygotes by measuring micronucleus induction in lymphocytes exposed to low dose rate (LDR) γ-irradiation in the $G_0$ phase of the cell cycle (Scott et al, 1996). The use of LDR exposure is believed to amplify small differences in repair capacity (Jones et al, 1995). An alternative method of amplification is by delaying mitogenic stimulation of irradiated $G_0$ cells to allow time for the expression of differential repair (Little and Nagawawa, 1985). Delaying stimulation for a few hours results in a reduction of chromosome damage in lymphocytes, presumably reflecting repair of lesions that lead to aberrations (Jones, 1995).

In the present study, we have compared sensitivity to radiation-induced micronucleus (MN) induction of healthy controls with that of an unselected series of women with breast cancer. Lymphocytes were exposed to high or low dose rate irradiation in $G_0$ and mitogen stimulated 6 h later. In a study to assess the reproducibility of the assay, we performed six repeat experiments on each of five healthy controls.

### MATERIALS AND METHODS

### Selection of controls

The controls were selected from normal volunteers including some spouses of the breast cancer patients. In the study of assay reproducibility, we performed a series of experiments on a panel of five control individuals who agreed to give blood samples on six occasions over a period of 6 months. The five control subjects comprised two men and three women and ranged in age from 23 to 46 years. These individuals are hereafter referred to as 'controls'. A further 42 normal volunteers were each tested on a single occasion and are subsequently referred to as 'normals'. Nineteen of the normals were tested in a planned series in which one of the five controls, one or two normals and one or two cancer patients were tested in parallel in each experiment (series A). A further series (series B) of 23 normals was also tested and as there were no differences in results between the two series they have been pooled for analysis. Normals comprised 28 women and 14 men between 23 and 72 years of age.

### Selection of breast cancer cases

The breast cancer cases were all attending the Christie Hospital for post-operative radiotherapy after a wide local excision with breast conservation 8–12 weeks earlier. They were either stage T1 ($n$ = 27) or T2 ($n$ = 12) and were all node negative apart from six

© Cancer Research Campaign 1998

**Figure 1** Micronucleus (●) and binucleate cell (□) frequencies in lymphocytes of a normal donor exposed to 3.5 Gy HDR irradiation and harvested between 68 and 94 h after PHA stimulation

**Figure 2** Micronucleus frequencies in five normal donors each tested six times for sensitivity to HDR (■) or LDR (□) irradiation (3.5 Gy). Dashed lines indicate the mean values of the six repeat tests patients, who were stage N1 (UICC TNM stage). They ranged in age from 35 years to 70 years. None had any evidence of metastatic disease or had any exposure to cytotoxic chemotherapy. Blood samples were taken for the micronucleus assay before radiotherapy commenced as localized radiotherapy may affect the in vitro radiosensitivity of lymphocytes (Rigaud et al, 1990). Their age, grade of tumour, menopausal status, tamoxifen intake, smoking history and family history of breast cancer were recorded.

### Micronucleus assay

The protocol was basically that of Fenech and Morley (1985) with optimization of conditions based upon our previous experience of $G_0$ chromosomal radiosensitivity assays (e.g. Jones, 1995; Jones et al, 1995; Scott et al, 1996).

Blood samples were obtained (with consent and ethical approval) by venepuncture, using sodium heparin as an anticoagulant. The blood was always stored overnight at room temperature and then diluted 1:10 with tissue culture medium (RPMI-1640, 20% fetal calf serum and 4 mM L-glutamine) prewarmed to 37°C in a 5% carbon dioxide atmosphere. The same serum batch was used throughout the investigations. For the HDR assay, two 5-ml aliquots of the blood in medium were placed in tissue culture flasks (Falcon T25). The flasks were kept for 1 h at 37°C before one flask was irradiated with 3.5 Gy $^{137}Cs$ γ-rays (dose rate 1.0 Gy $min^{-1}$) and the other sham irradiated. For the LDR assay, samples were divided between two tissue culture 24-well plates (Falcon), 2 ml per well, two wells per sample, one plate to be irradiated with 3.5 Gy $^{137}Cs$ γ-rays (dose rate 0.15 cGy $min^{-1}$, total exposure time 38.8 h) and the other to act as a sham-irradiated control. Throughout the irradiation period, all samples were maintained at 37°C in 5% carbon dioxide atmosphere in a purpose-built irradiation facility.

After irradiation, the protocol was the same for both assays. After a period of 6 h the lymphocytes were stimulated with the mitogen phytohaemagglutinin (PHA; Murex, HA15, final concentration 10 μg $ml^{-1}$). Twenty-four hours after stimulation, the cytokinesis-blocking agent cytochalasin-B (Sigma chemicals) was added to the cultures at a final concentration of 6 μg $ml^{-1}$. First generation post-mitotic cells could subsequently be identified as binucleated cells (Fenech and Morley, 1985). In a preliminary experiment we investigated the effect of harvesting time on MN yields because of previous conflicting reports (Lee et al, 1994; Kligerman and King, 1995). We found a steep increase in MN frequencies between 68 and 76 h and a plateau level thereafter (Figure 1). We therefore chose a 90 h sampling time for these studies, which has the additional advantage of a higher yield of binucleate cells for MN analysis than the usual sampling time of 72 h (Figure 1).

**Table 2** Radiation-induced micronucleus yields in normal and breast cancer patients (Figures 3 and 4)

| | | Micronuclei per 100 cells ± s.d. | | |
|---|---|---|---|---|
| **Group** | **Dose rate** | **Men** | **Women** | **All cases** |
| Normal (*n* = 42) | HDR | 44.6 ± 7.0 (*n* = 14) | 46.4 ± 9.8 (*n* = 28) | 45.8 ± 8.9 |
| | LDR | 23.1 ± 3.6 | 20.8 ± 5.9 | 22.2 ± 5.3 |
| Breast cancer cases (*n* = 39) | HDR | – | 60.7 ± 9.6 | 60.7 ± 9.6[a] |
| | LDR | – | 26.4 ± 7.9 | 26.4 ± 7.9[a] |

[a]Significant difference (Mann–Whitney *U*-test) between normal subjects and breast cancer patients; high dose rate (HDR), $P < 0.001$; low dose rate (LDR), $P = 0.003$.

© Cancer Research Campaign 1998

For harvesting, the cultures were centrifuged at 1000 r.p.m. for 5 min, the supernatant aspirated and the cells resuspended in 0.075 M potassium chloride at 4°C to lyse the erythrocytes. After exactly 2 min, the cells were centrifuged (1000 r.p.m. for 5 min), the supernatant rapidly aspirated and the cell pellet resuspended in approximately 0.5 ml of remaining solution. To this was added 5 ml of fixative (methanol–acetic acid, 25:1). All harvesting procedures and reagents were at ambient temperature unless otherwise stated. After further centrifuging and changing the fixative, they were stored at 4°C overnight. The samples were allowed to reach ambient temperature, centrifuged and most of the supernatant discarded. The cells were resuspended in approximately 0.5 ml of the remaining fixative, dropped gently onto slides and air-dried before staining (Leishman's full strength for 3 min, 30% stain for 12 min, diluted with buffer at pH 6.8), washing three times with pH 6.8 buffer, and drying and mounting.

All slides were coded, randomized to ensure anonymity of samples and analysed by one observer at a magnification of × 500, using a × 25 oil immersion objective. For series A the proportion of mono-, bi- and polynucleated cells was recorded in 100 consecutive cells, and for series A and B the micronucleus frequency was recorded in 100 consecutive binucleate cells. The criteria for scoring micronuclei were broadly similar to those of Countryman and Heddle (1976). To calculate the radiation induced micronucleus frequency per 100 binucleate cells (hereafter designated as the induced MN yield), the spontaneous micronucleus frequency in the unirradiated sample was subtracted from that obtained for the irradiated sample.

### Statistical analysis

A one-way analysis of variance was used to quantify the inter- and intraindividual variance within the assay.

Groups were compared using non-parametric Mann–Whitney *U*-tests and Kruskall–Wallis tests. Correlations between continuous variables (e.g. MN yields and age) were tested using Spearman's Rank correlation tests, and it is this correlation coefficient that is quoted here.

The numbers of sensitive patients were determined by selecting an arbitrary cut-off of the mean + 2 s.d. of the normal population (this would be an approximate 95% confidence limit if the population were large and the values normally distributed).

### RESULTS

### Assay reproducibility

The five normal controls were each tested six times. The mean induced MN scores were 44.0 ± 4.8 at HDR and 19.2 ± 3.6 at LDR. The coefficient of variation within individuals, calculated by one-way analysis of variance was 9% at HDR and 18% at LDR, indicating good assay reproducibility (Figure 2) particularly at HDR, such that there were significant interindividual differences at HDR ($P = 0.006$) but not at LDR ($P = 0.36$).

### Comparison of breast cancer cases with normals

The breast cancer cases ($n = 39$) were significantly more radiosensitive than the normals ($n = 42$) at HDR ($P < 0.001$). At LDR the difference was smaller but statistically significant ($P = 0.003$). Results are presented in Table 2 and Figures 3 and 4. The proportion of breast cancer cases with HDR MN yields that were greater than the mean + 2 s.d. of the normals was 31% (12 out of 39). The corresponding figure for normals was 5% (two out of 42), compared with an expectation of 2.5% for a normally distributed population. This 'cut-off' point is shown in Figure 3. At LDR, 15% (six out of 39) of the cancer patients and 5% (2 out of 42) of the normals were sensitive using the mean + 2 s.d. cut-off. There was only a weak correlation between HRD and LDR responses in patients [$r$ = (correlation coefficient) 0.18, $P = 0.29$] and normals ($r = 0.33$, $P = 0.04$). In part, this may be due to the experimental variability of the LDR assay but may also indicate that the HDR

**Figure 3** Micronucleus frequencies in normal subjects and breast cancer patients after HDR irradiation. Dashed lines represent mean values. The solid line indicates the 'cut-off' point for sensitivity, i.e. mean of normal + two s.d.s

**Figure 4** Micronucleus frequencies in normal subjects and breast cancer patients after LDR irradiation. See legend to Figure 3 for further details

© Cancer Research Campaign 1998

**Figure 5** Relationships between induced MN yields (HDR) and age for normal subjects and breast cancer patients. Dashed and dotted regression lines are for normal subjects and cancer patients respectively. ●, Normal; ○ breast carcinoma

**Table 3** Cell proliferation indices in normal and breast cancer patients

| | | Post-mitotic cells ± s.d. (%) | |
|---|---|---|---|
| **Group** | **Dose rate** | **Binucleate** | **Bi- + polynucleate** |
| Normal subjects | HDR | 61.1 ± 6.6 | 73.3 ± 6.3 |
| ($n$ = 19[a]) | HDR control[b] | 60.8 ± 8.6 | 72.6 ± 7.7 |
| | LDR | 66.0 ± 7.7 | 75.5 ± 6.5 |
| | LDR control[b] | 63.9 ± 16.1 | 74.5 ± 17.4 |
| Breast cancer | HDR | 59.7 ± 6.7 | 72.3 ± 6.5 |
| cases ($n$ = 39) | HDR control[b] | 63.6 ± 7.9 | 75.4 ± 6.7 |
| | LDR | 64.8 ± 8.1 | 74.5 ± 7.8 |
| | LDR control[b] | 64.7 ± 6.3 | 76.3 ± 6.4 |

[a]Cell proliferation was studied only in series A. [b]Unirradiated control samples were run in parallel with both the HDR and LDR samples.

and LDR assays are detecting different mechanisms of chromosomal radiosensitivity.

Spontaneous MN yields in breast cancer cases (1.6 ± 1.2; 200 cells scored from each of 39 cases) were significantly higher than in normals (0.7 ± 0.9; 200 cells from 42 individuals), $P = 0.001$ in a Mann–Whitney $U$-test.

### Potential confounding factors

There was a significant difference between the average age of the breast cancer patients (58.5 ± 7.4) and the normal subjects (47.8 ± 13.4, $P < 0.001$), and because of this there appears to be an increase in induced MN with age after HDR exposure when data for patients and normal subjects are combined (HDR, $r = 0.30$, $P = 0.007$, LDR, $r = 0.11$, $P = 0.33$). However, there was no age effect for cancer patients (HDR, $r = 0.05$, $P = 0.75$, LDR, $r = 0.04$, $P = 0.81$) or normals (HDR, $r = -0.06$, $P = 0.71$, LDR, $r = -0.09$, $P = 0.57$) when analysed separately (Figure 5), which suggests that the differences observed between breast cancer patients and normal subjects is not due to an age bias.

Among the normals there was no significant difference in response between men and women (Table 2), although the distribution of the sexes was biased towards women ($n$ = 28, men $n$ = 14).

Within the breast cancer group there was no significant correlation between induced MN scores (at HDR or LDR) and stage or grade of tumour, tamoxifen intake, menopausal status or smoking history.

Only three patients had any blood relatives with breast cancer. Each had one affected first degree relative. The three tested patients had HDR MN yields of 61, 52 and 91, respectively, with the last being the most sensitive of the 39 patients tested and the other two being well within the normal range for breast cancer cases (Fig. 3) and below the cut-off point for normal donors.

### Cell proliferation

At the 90 h harvesting time approximately 60% of cells were binucleate and 75% were either bi- or polynucleate (having undergone one or more mitotic divisions since stimulation), regardless of whether the samples were from normals or breast cancer patients or whether or not they were irradiated (Table 3). In our previous study using a 72 h sampling time (Scott et al, 1996), the corresponding figures for normals were approximately 45% and 50%, respectively, for unirradiated samples and significantly less after irradiation, probably indicating mitotic delay. The use of a later sampling time has the advantage of a greater yield of binucleate cells for MN analysis, and by 90 h the cells appear to have recovered from mitotic delay.

### DISCUSSION

Our use of LDR irradiation in these studies was based upon our previous observation that, in $G_0$ lymphocytes, discrimination between the chromosomal radiosensitivity of normals and A-T heterozygotes was possible only at LDR and not at HDR (Jones et al, 1995; Scott et al, 1996). However, in the previous studies PHA stimulation of HDR-irradiated lymphocytes occurred shortly after irradiation, whereas here we have allowed a 6-h interval for repair. It is likely that this modification of the HDR protocol, and the fact that there is less experimental variability at HDR than at LDR, has resulted in a better discrimination between normals and breast cancer cases at HDR. Indeed, we have now been able to discriminate between normals and A-T heterozygotes using this HDR protocol (unpublished observations). However, the rather weak correlation between HDR and LDR responses that we have seen in the present study suggests that these assays may be detecting different mechanisms of chromosomal radiosensitivity, and that some breast cancer cases and normals are defective in the 'HDR mechanism' and not in the 'LDR mechanism' and vice versa. We are further investigating this question in studies of larger numbers of individuals.

The proportion of breast cancer cases that were sensitive in the HDR assay (31%) is similar to that found with our $G_2$ method (42%, Scott et al, 1994). However, the exact proportion of sensitive cases is very dependent on the level of cut-off used. The present choice of the mean + 2 s.d. of normals is arbitrary but reasonable. Larger studies are required to more accurately define this threshold. We are now investigating the correlation between $G_0$/MN and $G_2$ sensitivity in the same individuals. Already, from preliminary studies, it is evident that there are patients who are $G_2$ sensitive but not $G_0$ /MN sensitive and vice versa.

As described earlier, there are clearly several different mechanisms underlying elevated chromosomal radiosensitivity. Even within breast cancer cases there may be three pathways detected

© Cancer Research Campaign 1998

by different assays ($G_2$, HDR/MN, LDR/MN). If enhanced chromosomal radiosensitivity is indeed indicative of cancer predisposition, it follows that no single assay will detect all 'at-risk' individuals. Our results to date suggest that the proportion of breast cancer patients sensitive in one or more of our three assays will considerably exceed 50%. This figure is not inconsistent with recent epidemiological studies suggesting genetic predisposition, via low-penetrance genes, in a high proportion of breast cancer cases (Teare et al, 1994; Chen et al, 1995; Houlston et al, 1996).

Many further studies will be required before chromosomal radiosensitivity assays could confidently be used to predict cancer predisposition in the general population. For example, it has to be shown that elevated chromosomal radiosensitivity is a heritable trait (family studies), that it is specific for cancer (studies of diseases other than cancer, both heritable and non-heritable), that it is not a consequence of physical and psychological stresses associated with diagnosis or treatment (follow-up studies after treatment) and that it correlates with the degree of genetic predisposition (studies of common cancers other than breast). We are investigating all of these possibilities. Ultimately, it must be shown that healthy individuals with elevated sensitivity are at greater risk of cancer than those of normal sensitivity (prospective studies). Knight et al (1993) have indirectly addressed this last question using the $G_2$ assay and have demonstrated a stronger family history of cancer in chromosomally radiosensitive cases, which is an encouraging observation.

The ability to identify individuals within the general population at increased risk of common cancers could lead to more effective use of resources in targeting individuals for intensive screening. Cytogenetic assays of radiosensitivity may have an important role in selecting these individuals.

### ACKNOWLEDGEMENTS

Funding for these studies was provided by the Cancer Research Campaign. In addition, we would like to thank Nigel Blakey, Chris Unsworth, Jim Caldwell and John Folds who completed a sponsored ascent of Mount Kilimanjaro, Kenya, and donated the proceeds to the Paterson Institute for construction of the low dose rate irradiation facility used in these studies and designed/ constructed by Dr Donald Allan and the Physics and Instrumentation Department.

### REFERENCES

Böcker W, Muller WU and Streffer C (1995) Image processing algorithms for the automated micronucleus assay in binucleated human lymphocytes. *Cytometry* **19**: 283–294

Chen PL, Sellers TA, Rich SS, Potter JD and Folsom AR (1995) Segregation analysis of breast cancer in a population-based sample of postmenopausal probands. *Genet Epidemiol* **12**: 401–415

Countryman PI and Heddle JA (1976) The production of micronuclei from chromosome aberrations in irradiated cultures of human lymphocytes. *Mutat Res* **41**: 321–332

Countryman PI, Heddle JA and Crawford E (1977) The repair of X-ray induced chromosomal damage in trisomy 21- and normal diploid lymphocytes. *Cancer Res* **37**: 52–58

Debauche DM, Pal GS and Stanley WS (1990) Enhanced $G_2$ chromatid radiosensitivity in dyskeratosis congenita fibroblasts. *Am J Hum Genet* **46**: 350–357

Duckworth-Rysiecki G and Taylor AM (1985) Effects of ionising radiation on cells from Fanconi's anaemia patients. *Cancer Res* **45**: 416–420

Easton DF (1994) Cancer risks in A-T heterozygotes. *Int J Radiat Biol* **64** (suppl): 177–182

EL-Zein R, Shaw P, Tyring SK and Au WW (1995) Chromosomal radiosensitivity of lymphocytes from skin cancer-prone patients. *Mutat Res* **335**: 143–149

Featherstone T, Taylor AM and Harnden DG (1983) Studies on the radiosensitivity of cells from patients with basal cell naevus syndrome. *Am J Hum Genet* **35**: 58–66

Fenech M and Morley AA (1985) Measurements of micronuclei in lymphocytes. *Mutation Res* **149**: 475–483

Ford D and Easton DF (1996) The breast-ovarian cancer syndrome and BRCA1. In *Genetic Predisposition to Cancer*, Eeles RA, Ponder BAJ, Easton DF and Horwich A (eds), pp. 239–252. Chapman & Hall: London

Gibbons B, Scott D, Hungerford JL, Cheung KL, Harrison C, Attard-Montalto S, Evans M, Birch, JM and Kingston JE (1995) Retinoblastoma in association with the chromosome breakage syndromes Fanconi's anaemia and Bloom's syndrome: clinical and cytogenetic findings. *Clin Genet* **47**: 311–317

Goldgar DE, Stratton MR and Eeles RA (1996) Familial breast cancer. In *Genetic Predisposition to Cancer*, Eeles RA, Ponder BAJ, Easton DF and Horwich A (eds), pp. 227–238. Chapman & Hall: London

Heddle JA, Krepinsky AB and Marshall RR (1983) Cellular sensitivity to mutagens and carcinogens in the chromosome-breakage and other cancer-prone syndromes. In *Chromosome Mutation and Neoplasia*, German J (ed), pp. 203–234. Liss: New York

Heddle JA, Lue CB, Saunders EF and Benz RD (1978) Sensitivity to five mutagens in Fanconi's anaemia as measured by the micronucleus method. *Cancer Res* **38**: 2983–2988

Heim S and Mitelman F (1996) *Cancer Cytogenetics*. Wiley-Liss: New York

Higurashi M and Conen PE (1973) *In vitro* chromosomal radiosensitivity in 'chromosomal breakage syndromes'. *Cancer* **32**: 380–383

Hittelman WN and Pandita TK (1994) Possible role of chromatin alteration in the radiosensitivity of ataxia-telangiectasia. *Int J Radiat Biol* **66**(suppl.): 109–113

Houlston RS and Peto J (1996) Genetics and the common cancers. In *Genetic Predisposition to Cancer*, Eeles RA, Ponder BAJ, Easton DF and Horwich A (eds), pp. 208–226. Chapman and Hall: London

Jaspers NG, Gatti RA, Baan C, Linssen PC and Bootsma D (1998) Genetic complementation analysis of ataxia-telangiectasia and Nijmegen breakage syndrome: a survey of 50 patients. *Cytogenet Cell Genet* **49**: 259–263

Jones L A, Scott D, Cowan R and Roberts SA (1995) Abnormal radiosensitivity of lymphocytes from breast cancer patients with excessive normal tissue damage after radiotherapy: chromosome aberrations after low dose-rate irradiation. *Int J Radiat Biol* **67**: 519–528

Jones LA (1995) Chromosomal radiosensitivity as an indicator of cancer predisposition. PhD thesis, University of Manchester, UK.

Kerr B, Ashcroft G, Scott D, Horan M, Fergusen MWJ and Donnai D (1996) Rothmund-Thomson syndrome: two cases reports reveal heterogenous cutaneous abnormalities, an association with genetically programmed ageing changes and elevated chromosomal radiosensitivity. *J Med Genet* **33**: 928–934

Kinsier KW and Vogelstein B (1997) Cancer-susceptibility genes. Gatekeepers and caretakers. *Nature* **386**: 761–763

Kligerman AD and King SC (1995) Frequency of micronucleated-binucleated lymphocytes is not significantly affected by harvest time following $G_0$ exposure to X-irradiation. *Int J Radiat Biol* **68**: 19–23

Knight RD, Parshad R, Price FM, Tarone RE and Sanford KK (1993) X-ray induced chromatid damage in relation to DNA repair and cancer incidence in family members. *Int J Cancer* **54**: 589–593

Kuhn EM (1980) Effects of X-irradiation in $G_1$ and $G_2$ on Bloom's syndrome and normal chromosomes. *Hum Genet* **54**: 335–341

Lavin MF, Bennett I, Ramsey J, Gardiner RA, Seymour GJ, Farrell A and Walsh M (1994) Identification of a potentially radiosensitive subgroup among patients with breast cancer. *J Natl Cancer Inst* **86**: 1627–1634

Lavin MF, LE Poidevin P and Bates P (1992) Enhanced levels of radiation-induced $G_2$ phase delay in ataxia-telangiectasia heterozygotes. *Cancer Genet Cytogenet* **60**: 183–187

Lee TK, Wiley AL, Esinhart JD and Blackburn LD (1994) Radiation dose-dependent variations in micronuclei production in cytochalasin B-blocked human lymphocytes. *Teratog Carcinog Mutagen* **14**: 1–12

Little JB and Nagasawa H (1985) Effect of confluent holding on potentially lethal damage repair, cell cycle progression and chromosome aberrations in human normal and ataxia-telangiectasia fibroblasts. *Radiat Res* **101**: 81–93

Morten JE, Harnden DG and Taylor AM (1991) Chromosome damage in- $G_0$ X-irradiated lymphocytes from patients with hereditary retinoblastoma. *Cancer Res* **41**: 3635–3638

Mozdarani H and Bryant PE (1989) Kinetics of chromatid aberrations in $G_2$ ataxia-telangiectasia cells exposed to x-rays and ara-A. *Int J Radiat Biol* **55**: 71–84

Natarajan AT, Vyas RC, Darroudi F, Mullenders LHF and Zdzienicka MZ (1990) DNA lesions, DNA repair and chromosomal aberrations. In *Chromosomal Aberrations: Basic and Applied Aspects*, Obe G and Natarajan AT (eds), pp. 31–40. Springer: Berlin

Parshad R, Sanford KK and Jones GM (1983) Chromatid damage after $G_2$ phase x-irradiation of cells from cancer prone individuals implicates deficiency in DNA repair. *Proc Natl Acad Sci USA* **80**: 5612–5616

Parshad R, Price FM, Bohr VA, Cowans KH, Zujewski JA and Sanford KK (1996) Deficient DNA repair capacity, a predisposing factor in breast cancer. *Br J Cancer* **74**: 1–5

Parshad R, Price FM, Pirollo KF, Chang EH and Sanford KK (1993) Cytogenetic response to $G_2$ phase X irradiation in relation to DNA repair and radiosensitivity in a cancer-prone family with Li–Fraumeni syndrome. *Radiat Res* **136**: 236–240

Preston RJ (1980) DNA repair and chromosome aberrations: the effect of cytosine arabinoside on the frequency of chromosome aberrations induced by radiation and chemicals. *Teratog Carcinog Mutagen* **1**: 147–159

Price FM, Parshad R, Tarone RE and Sanford KK (1991) Radiation-induced chromatid aberrations in Cockayne syndrome and xeroderma pigmentosum group C fibroblasts in relation to cancer predisposition. *Cancer Genet Cytogenet* **577**: 1–10

Rigaud O, Guedeney G, Duranton I, Leroy A, Doloy MT and Magdelenat H (1990) Genotoxic effects of radiotherapy and chemotherapy on the circulating lymphocytes of breast cancer patients. I. Chromosome aberrations induced *in vivo*. *Mutat Res* **242**: 17–23

Sanford KK, Parshad R, Price FM, Tarone RE and Benedict WF (1996) Cytogenetic responses to $G_2$ phase X-irradiation of cells from retinoblastoma patients. *Cancer Genet. Cytogenet* **88**: 43–48

Sanford K, Tarone RE, Parshad R, Tucker MA, Greene MH and Jones GM (1987) Hypersensitivity to $G_2$ chromatid radiation damage in familial dysplastic naevus syndrome. *Lancet* **2**: 1111–1116

Sanford KK, Parshad R, Gantt RE, Tarone RE, Jones GM and Price FM (1989) Factors affecting and significance of $G_2$ chromatin radiosensitivity and predisposition to cancer. *Int J Radiat Biol* **55**: 963–98

Sanford KK, Parshad R, Price FM, Jones GM, Tarone RE, Eierman L, Hale P and Waldmann TA (1990) Enhanced chromatid damage in blood lymphocytes after $G_2$ phase x irradiation, a marker of the ataxia-telangiectasia gene. *J Natl Cancer Inst* **82**: 1050–1054

Sasaki MS, Tonomura A and Matsubara S (1970) Chromosome constitution and its bearing on the chromosomal radiosensitivity in man. *Mutat Res* **10**: 617–633

Schwartz JL, Jordan R, Sedita BA, Swenningson MJ, Banáth JP and Olive PL (1995) Differential sensitivity to cell killing and chromosome mutation induction by gamma rays in two human lymphoblastoid cell lines derived from a single donor: possible role of apoptosis. *Mutagenesis* **10**: 227–233

Scott D, Hu Q, and Roberts SA (1996) Dose-rate sparing for micronucleus induction in lymphocytes of controls and ataxia-telangiectasia heterozygotes exposed to $^{60}Co$ $\gamma$-irradiation *in vitro*. *Int J Radiat Biol* **70**: 521–527

Scott D, Spreadborough A, Levine E and Roberts SA (1994) Genetic predisposition to breast cancer. *Lancet* **344**: 1444

Taalman RD, Jaspers NG, Scheres JM, De Wit J and Hustinx TW (1983) Hypersensitivity to ionising radiation, *in vitro*, in a new chromosomal breakage disorder, the Nijmegen breakage syndrome. *Mutat Res* **112**: 23–32

Taalman RD, Hustinx TW, Weemaes CM, Seemanova E, Shmidt A, Passarge E and Scheres JM (1989) Further delineation of the Nijmegen breakage syndrome. *Am J Med Genet* **32**: 425–431

Takeshita T, Higurashi M, Ariizumi Shibusawa C, Shimizu K, Iijima S, Yamagata Z, Asaka A, Morimoto K, Ishibashi Y and Otsuka F (1994) Elevated chromosome aberration frequency after X-ray exposure of cultured fibroblasts derived from patients with porokeratosis. *Cancer Genet Cytogenet* **73**: 161–164

Taylor AM, Metcalfe JA, Oxford JM and Harnden DG (1976) Is chromatoid-type damage in ataxia telangiectasia after irradiation at $G_0$ a consequence of defective repair? *Nature* **260**: 441–443

Teare MD, Wallace SA, Harris M, Howell A and Birch JM (1994) Cancer experience in the relatives of an unselected series of breast cancer patients. *Br J Cancer* **10**: 102–111

Verhaegen F, Vral A, Seutjens J, Schipper NW, De Ridder L and Thierens H (1994) Scoring of radiation-induced micronuclei in cytokinesis-blocked human lymphocytes by automated image analysis. *Cytometry* **17**: 119–127

Vorechovsky I, Scott D, Haeney MR and Webster D (1993) Chromosomal radiosensitivity in common variable immune deficiency. *Mutat Res* **290**: 255–264

Waghray DE, Al-Sedairy S, Ozand PT and Hannan MA (1990) Cytogenetic characterisation of ataxia-telangiectasia (AT) heterozygotes using lymphoblastoid cell lines and chronic irradiation. *Human Genet* **84**: 532–534

Wang L, Cui L, Lord BI, Roberts SA, Potten CS, Hendry JH and Scott D (1996) Gamma-ray-induced cell killing and chromosome abnormalities in the bone marrow of p53 deficient mice. *Radiation Res* **196**: 259–266

Ward JF (1994) The complexity of DNA damage: relevance to biological consequences. *Int J Radiat Biol* **66**: 427–432

Watanabe R, Ishibashi Y and Otsuka F (1990) Chromosomal instability and cellular hypersensitivity to X-irradiation of cultured fibroblasts derived from porokeratosis patients' skin. *Mutat Res* **230**: 273–278

Williams KJ, Boyle JM, Birch JM, Norton JD and Scott D (1997) Cell cycle arrest in Li–Fraumeni syndrome: A mechanism of cancer predisposition? *Oncogene* **14**: 277–282

© Cancer Research Campaign 1998